# Development and pilot testing of an informed consent video for patients with limb trauma prior to debridement surgery using a modified Delphi technique

**DOI:** 10.1186/s12910-017-0228-3

**Published:** 2017-11-29

**Authors:** Yen-Ko Lin, Chao-Wen Chen, Wei-Che Lee, Tsung-Ying Lin, Liang-Chi Kuo, Chia-Ju Lin, Leiyu Shi, Yin-Chun Tien, Yuan-Chia Cheng

**Affiliations:** 1 Division of Trauma and Surgical Critical Care, Department of Surgery, Kaohsiung Medical University Hospital, Kaohsiung Medical University, Kaohsiung, Taiwan; 2 Department of Medical Humanities and Education, College of Medicine, Kaohsiung Medical University, Kaohsiung, Taiwan; 3 Department of Emergency Medicine, College of Medicine, Kaohsiung Medical University, Kaohsiung, Taiwan; 4 College of Nursing, Kaohsiung Medical University, Kaohsiung, Taiwan; 5 Department of Medical Research, Kaohsiung Medical University Hospital, Kaohsiung Medical University, Kaohsiung, Taiwan; 6 Department of Health Policy and Management, Bloomberg School of Public Health, Johns Hopkins University, Baltimore, Maryland 21205 USA; 7 Department of Orthopedics, Kaohsiung Medical University Hospital, Kaohsiung Medical University, Kaohsiung, Taiwan; 8 Department of Orthopedics, College of Medicine, Kaohsiung Medical University, Kaohsiung, Taiwan

**Keywords:** Informed consent, Educational video, Trauma, Emergency department, Delphi technique

## Abstract

**Background:**

Ensuring adequate informed consent for surgery in a trauma setting is challenging. We developed and pilot tested an educational video containing information regarding the informed consent process for surgery in trauma patients and a knowledge measure instrument and evaluated whether the audiovisual presentation improved the patients’ knowledge regarding their procedure and aftercare and their satisfaction with the informed consent process.

**Methods:**

A modified Delphi technique in which a panel of experts participated in successive rounds of shared scoring of items to forecast outcomes was applied to reach a consensus among the experts. The resulting consensus was used to develop the video content and questions for measuring the understanding of the informed consent for debridement surgery in limb trauma patients. The expert panel included experienced patients. The participants in this pilot study were enrolled as a convenience sample of adult trauma patients scheduled to receive surgery.

**Results:**

The modified Delphi technique comprised three rounds over a 4-month period. The items given higher scores by the experts in several categories were chosen for the subsequent rounds until consensus was reached. The experts reached a consensus on each item after the three-round process. The final knowledge measure comprising 10 questions was developed and validated. Thirty eligible trauma patients presenting to the Emergency Department (ED) were approached and completed the questionnaires in this pilot study. The participants exhibited significantly higher mean knowledge and satisfaction scores after watching the educational video than before watching the video.

**Conclusions:**

Our process is promising for developing procedure-specific informed consent and audiovisual aids in medical and surgical specialties. The educational video was developed using a scientific method that integrated the opinions of different stakeholders, particularly patients. This video is a useful tool for improving the knowledge and satisfaction of trauma patients in the ED. The modified Delphi technique is an effective method for collecting experts’ opinions and reaching a consensus on the content of educational materials for informed consent. Institutions should prioritize patient-centered health care and develop a structured informed consent process to improve the quality of care.

**Trial registration:**

The ClinicalTrials.gov Identifier is NCT01338480. The date of registration was April 18, 2011 (retrospectively registered).

## Background

The doctrine of informed consent has been recognized as a principal ethical foundation of medicine for the last five decades. Informed consent is also a legal prerequisite in contemporary medicine. Informed consent encourages patients to be actively engaged in their health decision-making process concerning treatment [1–4].

Because most situations that occur in emergency settings involve time constraints, emotional stress and physical pain due to sudden injury in patients, the patients and their families often experience difficulty in understanding all the important information that is essential for providing informed consent [1, 2, 5]. Some authors have reported that recall is variable and may be poor when patients attempt to remember the consent process during acute illness. In fact, some patients are unable to recall the process at all [6]. Poor recall is particularly evident in trauma patients [7–10]. Because of the unique conditions in emergency situations, the informed consent process is one of the most profound and emotional challenges for trauma patients and their families. During the traditional consent process, trauma patients have difficulty in retaining the vast amount of information presented to them. These patients are often unable to imagine how the surgery would proceed. Consequently, the patients and their families might not provide appropriate consent because they are unaware of the risks and complications.

Audiovisual presentations are promising tools for educating patients in emergency settings [11, 12]. An audiovisual presentation augments the routine informed consent process. A cooperative effort by healthcare providers should convey critical information more effectively using more than one method and help patients and family members obtain adequate knowledge for making a rational treatment decision even under stressful conditions.

This study aimed to develop and pilot test an educational video containing information regarding the informed consent process for trauma patients undergoing surgery, develop and pilot test a knowledge measure instrument, and evaluate whether the audiovisual presentation improved the patients’ understanding of their procedure and aftercare and their satisfaction with the informed consent process. Our study presents a successful process for developing procedure-specific informed consent audiovisual aids that may be used in medical and surgical specialties.

## Methods

### Development of the educational video

The first stage of the study comprised the development of the educational video. We first considered the type of surgery or procedure for which to develop a video to educate trauma patients in an emergency setting. A video that could be used with all trauma patients would have been ideal. However, each surgery has unique procedures, risks, benefits, and alternatives, and a “one-size-fits-all” video that could apply to all trauma patients would have been difficult to develop. Hence, we opted to develop a video specific to one surgery or procedure. The criteria considered in prioritizing the type of surgery included 1) benefitting as many trauma patients as possible by selecting a surgery that most trauma patients might receive and 2) choosing a procedure that was not life or limb threatening because, in these situations, the patient might be sent to the operating room within minutes. Therefore, the final decision was to use debridement surgery for complicated limb wounds.

Next, we considered the content to be included in the development of the video. The content was developed according to the following procedure. A panel of experts was invited to participate. Based on the literature, we identified the procedures, risks, benefits, and alternatives for debridement surgery. A modified Delphi technique [13] was applied to collect the opinions of the experts, who contributed to the development of the video. The results from the experts were finalized, and the transcript for the video was revised. Most patients prefer to have more information when making medical decisions [14]. However, after conducting a survey in an emergency clinic for oral surgery, Degerliyurt et al. concluded that an overly thorough informed consent process could disclose too much information to the patients and become overwhelming; [15] therefore, the content of the video should contain the precise information that patients wish to know within the ethical principles and legal regulations. The expected length of the video was limited to 15 min.

After the transcript had been confirmed, we contracted a multimedia company to produce the video. Several different possibilities for displaying the video were available, including role-play by an actor, 2-dimensional (2D) or 3-dimensional (3D) graphics, or an interactive computer program. While an interactive computer program could be tailor-made for the patients and offer promising results, [16] it has the disadvantages of a higher cost and longer production time; therefore, using the 2D or 3D graphics was more appealing. Role-playing by actors may appear authentic but could result in patient discomfort while watching the video, and displaying the details of the surgical procedure may be difficult; therefore, a combination of the 2D and 3D graphics was chosen for the development of the video because the cost of the 3D graphics is higher than that of the 2D graphics. The developed video contained visual aids and an audio narration. The audio narration described the content displayed in the video. Subtitles and captions were added. After determining the details, a multimedia company was contracted to produce the video within a given time frame and according to our specifications. A preliminary version of the video was sent to the experts for review, and their comments and opinions were considered in revising the video to its final version.

### The expert panel

A modified Delphi technique was applied to reach a consensus among a panel of experts who were chosen to help develop the video content and choose the questions to measure the understanding of informed consent for debridement surgery in trauma patients. In this study, several experts from different fields with a variety of expertise, including trauma surgeons, plastic surgeons, nurses, informed consent experts, a lawyer, and patients who had previously received the surgery, were invited to participate in a Delphi round after agreeing to participate in this study. Each expert was chosen based on recommendations by two specialists from Kaohsiung Medical University’s Health Care System, which includes one tertiary medical center with more than 1600 beds and two metropolitan hospitals with more than 800 beds. The patients were recommended by nurse practitioners who worked in the plastic surgery ward.

### The modified Delphi technique

In our modified Delphi technique, during the first Delphi round, open-ended questions were not used to collect the experts’ opinions because such questions might make responding more difficult and thus decrease the experts’ response rate. Furthermore, because the third and fourth Delphi rounds were combined, the modified Delphi technique included only three rounds. Brooks reported that a three-round investigation might be sufficient for experts to reach a consensus [17].

### Survey of the experts

First, a transcript containing the informed consent information regarding the procedures, risks, complications, benefits, and alternatives to the surgery was developed and summarized based on reports from the literature [13, 18–24]. In addition to the informed consent information, the video content included the topics of how to choose the appropriate procedure, preparation for the surgery, anesthesia, and post-operative recovery and care. The experts were asked to provide their opinions on the items included in the draft transcript and indicate which items they considered important for trauma patients to know during the informed consent process. The questionnaire was sent to the experts by e-mail and returned when completed. A space was provided at the end of the questionnaire for the experts to include other comments.

After receiving the first-round questionnaire, the investigators revised the transcript according to the experts’ responses. The second-round questionnaire include all items and the additional comments from the experts. During the second round, the questionnaire was sent to the experts by e-mail, and the experts were asked to rank the importance and appropriateness of each item on a five-point Likert scale. After receiving and analyzing the results of the second-round questionnaire, the investigators concluded that a consensus was reached. The experts were then sent by e-mail an abstract including the consensus and the results, showing the minimum and maximum values, mean and median of each item, and were asked to compare their opinions with those of the other experts; the experts were allowed to change their responses if desired. The experts were then asked to complete a third-round questionnaire using the same ranking procedure. Consensus was defined as a mean score on each item of 3.75 or greater for importance and appropriateness. The differences in the experts’ ratings of each item between the second and third round were also compared.

### The knowledge measure instrument

Another aspect of the study was to develop a knowledge measure instrument. Based on the literature, no measurement instrument had been developed that could be applied to measure the understanding of trauma patients regarding informed consent for surgery. Thus, we developed a knowledge measure instrument specifically for this purpose.

The questionnaire measured patient demographics, including age, gender, and level of education. The questions measuring the patients’ knowledge of the surgery they were consenting to receive covered the content of the video, which was based on the experts’ consensus. Each question was weighted equally. The questions were written in a multiple-choice format. Twenty questions were developed and distributed to the panel of experts. The experts were asked to rate each item on a five-point scale from “strong agreement” to “strong disagreement”. The rating results were analyzed, and the top ranked 13 questions were selected for the instrument test.

The measurement instrument was tested on 10 subjects who had been selected from an Emergency Department (ED). Questions that were correctly answered by more than 85% of subjects and those that poorly correlated with the total scores were replaced. The results of the study were used to identify problematic questions, and the measurement instrument was then revised. The instrument was administered in the pilot study, and questions were further eliminated if the correct response rate had no statistical significance in the pilot study.

### Pilot study

Participants were enrolled as a convenience sample of adult trauma patients scheduled to receive wound debridement surgery. The following assumptions apply to the power analysis in this study: (a) the intervention increases the mean score on the measurement instrument by 10%; (b) the scores are normally distributed; (c) the standard deviation is 18; (d) the level of significance is 0.05 (*p* < 0.05); and (e) a two-tailed t-test is used. Given these assumptions, a sample size of 28 was needed to achieve 80% power with a significance of 0.05.

The participants had received information orally from their healthcare providers and completed the knowledge measure as a baseline before watching the educational video. The participants then watched the educational video illustrating the surgical procedure and its benefits, risks, and alternatives at their bedside on a laptop computer. After watching the video, the participants were asked to complete the knowledge measure again. Questions using a 5-point Likert scale were employed to evaluate the participants’ satisfaction with the educational video before and after the educational session.

### Data processing and statistical analysis

The data collected from the patients were identified only by a participant number without any information specific to the patient to protect the patients’ privacy and secure confidentiality. Descriptive statistics were performed to analyze the baseline characteristics of the participants. The means and standard deviations were calculated for normally distributed continuous variables, and proportions were calculated for categorical variables. The differences in the experts’ ratings of each item between the second and third rounds were compared using a Wilcoxon signed-rank test. McNemar’s exact test was performed to compare the correct response rate on the knowledge test for each question before and after watching the educational video. The mean scores before and after watching the educational video on the knowledge measure and the patients’ satisfaction were calculated and analyzed. The changes in knowledge between before and after watching the educational video were compared using the paired t*-*test, and the changes in the satisfaction ratings were compared using the Wilcoxon signed-rank test. All data analyses were performed using Stata version 10.0 (StataCorp, College Station, TX, USA).

The study protocol was approved by the Institutional Review Board of Kaohsiung Medical University Hospital before the study commenced. All patients in this study signed written informed consent prior to enrollment.

## Results

### Demographic characteristics of the experts

Sixteen experts from different fields with a variety of expertise, including trauma surgeons, nurses and nurse practitioners, members of the ethics committee, a lawyer, and patients who had previously undergone the surgery were invited to participate in a Delphi round after agreeing to participate in this study. The baseline characteristics of the experts are provided in Table 1. The most common age group for the experts was 30–39 years, and most experts had attained a college-level education.

**Table 1:** Baseline characteristics of the Delphi experts

Characteristic	No.	%
Specialty		
Physician	5	31.2
Nurse or nurse practitioner	5	31.2
Patient	4	25.0
Member of ethics committee	1	6.3
Lawyer	1	6.3
Age		
20–29	1	6.3
30–39	9	56.2
40–49	4	25.0
50–59	2	12.5
Gender		
Female	7	43.8
Male	9	56.2
Education		
College	10	62.5
Post-graduate	6	37.5

### Three-round Delphi process

The modified Delphi technique comprised three rounds conducted over a 4-month period. After the first round, the questionnaire items were revised and rephrased according to the experts’ suggestions. The results of the second and third rounds in terms of benefits, procedures, risks, post-operative complications, and alternatives are provided in Table 2. The experts allocated higher scores to the items in the categories of benefits and alternatives and most items in the categories of risks and post-operative complications. During the second round, the experts allocated lower scores to some items in the category of procedures (items 1.3, 1.4, 1.6, and 1.7), which mainly described the details of the surgical procedure and anesthesia, but they reached consensus in the third round. The results of the second and third rounds for post-operative wound care are provided in Table 3. The experts allocated higher scores to items describing the purpose, appropriate timing and frequency of ice packing and hot packing, and procedures for changing dressings. Items with significant differences between the second and third rounds were also identified. Many items (4.7.2, 4.7.3, 4.7.4, 4.7.6, and 4.7.7), mainly describing the symptoms of possible wound infection, had significant differences between the second and third rounds. The experts reached a consensus on each item after the three-round process.

**Table 2:** Delphi results regarding benefits, procedures, risks and complications, and alternatives

Category	Item No.	Item	Importance				Appropriateness			
			Mean	Median	Min	Max	Mean	Median	Min	Max
Benefit	1.1	Surgical debridement is a procedure for removing dead tissue and foreign bodies from wounds and the fastest and most effective way to clean wounds. Surgical debridement may prevent infection and improve the wound healing process.	4.56/4.63	5/5	3/3	5/5	4.38/4.31	4.5/4	3/3	5/5
Procedure	1.2	The procedure may be performed at the bedside or in the operating room if the wound is too deep, large, or involves important tissue, such as nerves, vessels or muscle, to decrease the possibility of wound infection and other complications.	4.5/4.69	5/5	2/4	5/5	4.75/4.63	5/5	4/3	5/5
	1.3	When local anesthesia is chosen, the surgeon will inject the medication to anesthetize the region where the procedure will be performed.	3.94/4.44	4/4.5	1/3	5/5	4.00/4.19	4/4	2/3	5/5
	1.4	Epidural anesthesia may be chosen to anesthetize the lower part of the body by injecting the medication into the lumbar spinal cord when the procedure will be performed in the lower part of the body.	3.63/3.94	4/4*	2/2	5/5	3.94/4.00	4/4	3/3	5/5
	1.5	General anesthesia blocks the feeling of pain throughout the body, and you may fall asleep during the surgical procedure.	4.00/4.31	4/4	3/4	5/5	4.19/4.13	4/4	3/3	5/5
	1.6	The surgeon will clean the wound and remove as many contaminants as possible with normal saline.	3.81/4.31	4/4	1/3	5/5	4.25/4.06	5/4	1/3	5/5
	1.7	The surgeon may use surgical instruments to repeatedly remove dead tissue until the wound is clean.	3.69/4.00	4/4	1/3	5/5	3.94/3.94	4/4	1/3	5/5
	1.8	When the procedure is finished, the surgeon will close the wound layer by layer. If the wound is not closed immediately, the wound will be cared for openly.	4.56/4.88	5/5*	4/4	5/5	4.56/4.50	5/5	3/3	5/5
	1.9	The timing of wound closure will depend on the injury mechanism, location of the wound, and possibility of wound infection.	4.44/4.63	5/5	3/4	5/5	4.56/4.69	5/5	3/4	5/5
	1.10	The skin will be closed by suture, adhesive tape, or staples and covered with a sterile gauze or dressing.	4.13/4.25	4/4	3/3	5/5	4.13/4.50	4/5	3/3	5/5
Risks and postoperative complications	2.1	When debridement is performed, deep tissues, such as vessels, tendons, and nerves, might be injured, and complications can include bleeding, tendon injury, nerve injury, postoperative range ofmotion limitation in the limbs, wound pain, permanent scarring, etc.	4.25/4.50	5/5	3/3	5/5	4.38/4.31	4.5/4	2/2	5/5
	2.2	Bacteria from the skin might affect the deep tissues and cause infection, and the rate of infection might differ depending on the cause of the injury, mechanism, and location of the wound.	3.69/4.13	4/4	2/3	5/5	4.00/3.69	4/4	2/2	5/5
	2.3	Pre-existing illnesses, such as diabetes mellitus and immune-compromised diseases, and using steroids, anti-immune drugs, and anti-coagulants might increase the risks of the procedure and postoperative complications.	4.94/4.81	5/5	4/4	5/5	4.81/4.69	5/5	4/4	5/5
	2.4	Smoking, poor nutrition, and poor circulation might increase the risks of the procedure and postoperative complications.	4.69/4.81	5/5	4/4	5/5	4.81/4.81	5/5	4/4	5/5
	2.5	Unforeseen disorders might occur, such as shock and arrhythmia.	4.81/4.81	5/5	4/4	5/5	4.63/4.88	5/5*	4/4	5/5
	2.6	Complicated wounds require regular trips to the clinic to decrease the complications.	4.56/5.00	5/5*	3/5	5/5	4.75/4.88	5/5	3/4	5/5
Alternative	3.1	Wound management might be performed in other ways, such as using a bio-artificial dressing to debride the wound, but this takes 2~4 weeks and has an increased risk of wound infection. If you have any questions concerning the treatment, please discuss them with your physician.	4.38/4.38	4.5/4	3/4	5/5	4.19/4.31	4/4	3/3	5/5

*p < 0.05

**Table 3:** Delphi results regarding wound care

Category	Item No.	Item	Importance				Appropriateness			
			Mean	Median	Min	Max	Mean	Median	Min	Max
Wound care	4.1	Ice packing over the wound is recommended for one to three days after injury, which can be performed for 10–15 min three to four times per day. Ice packing may help to stop the bleeding and alleviate swelling and pain. In the meantime, the injured limb should be elevated above the heart to alleviate swelling and discomfort, and over-activity of the injured limb should be avoided.	4.81/4.94	5/5	4/4	5/5	4.94/4.94	5/5	4/4	5/5
	4.2	Hot packing is recommended 3 days after injury to improve circulation and alleviate the swelling of the wound.	4.19/4.50	4/5	3/3	5/5	4.13/4.69	4/5*	3/4	5/5
	4.3	Changing the dressing is suggested 2 days after injury for wounds. Normal saline can be used to clean the wound. The dressing should be kept dry and can be changed daily after bathing.	4.44/4.63	5/5	2/3	5/5	4.50/4.81	5/5*	3/4	5/5
	4.4	Please follow the orders of your doctor and other professionals to care for your wound. For wound care, you may need:	4.81/5.00	5/5	4/5	5/5	4.69/4.69	5/5	4/4	5/5
	4.4.1	Two clean disposable gloves	4.13/4.38	4/4	3/4	5/5	4.19/4.25	4/4	3/3	5/5
	4.4.2	Normal saline	4.69/4.63	5/5	4/4	5/5	4.75/4.69	5/5	4/4	5/5
	4.4.3	Small gauze or sterile cotton swab to clean the wound	4.44/4.69	4.5/5	3/4	5/5	4.50/4.75	4.5/5	4/4	5/5
	4.4.4	Large gauze to cover the wound	4.31/4.69	4/5*	3/4	5/5	4.44/4.69	4/5*	4/4	5/5
	4.4.5	Adhesive tape	4.38/4.50	5/4.5	3/4	5/5	4.50/4.50	5/4.5	3/4	5/5
	4.5	Procedure for changing the dressing:								
	4.5.1	First, clean and wash your hands and put on the clean gloves. Then, remove the covered gauze from the wound.	4.31/4.69	4/5	3/4	5/5	4.56/4.38	5/4.5	3/3	5/5
	4.5.2	Observe the color and odor of the discharge from the wound on the gauze.	4.19/4.63	4/5	2/4	5/5	4.31/4.31	4/4	3/3	5/5
	4.5.3	If the gauze adheres to the wound, normal saline can be used to rinse the gauze, and then the gauze can be removed gently a few minutes later.	4.5/4.56	4.5/5	4/4	5/5	4.38/4.75	4/5*	4/4	5/5
	4.5.4	You may use normal saline to rinse the wound in addition to using a small gauze or sterile cotton swabs.	4.38/4.50	4/4.5	3/4	5/5	4.56/4.56	5/5	4/4	5/5
	4.5.5	The wound can be cleaned by moving a small gauze or a sterile cotton swab up and down or in and out circularly, and the gauze and sterile cotton swab should be placed into a zip bag after cleaning the wound.	4.69/4.50	5/4.5	4/4	5/5	4.31/4.38	4/4	4/4	5/5
	4.5.6	Each wound requires a new gauze or sterile cotton swab.	4.56/4.63	5/5	3/4	5/5	4.75/4.44	5/4.5*	4/3	5/5
	4.5.7	In principle, the skin area within 10 cm of the wound should be cleaned with the gauze or sterile cotton swab from up to down, and the used gauze or cotton swab should be placed into a zip bag.	4.00/4.00	4/4	2/3	5/5	4.56/4.00	5/4*	4/3	5/5
	4.5.8	After cleaning the wound, a sterile cotton swab can be used to remove any discharge from the wound surface.	4.00/4.19	4/4	3/3	5/5	4.25/4.31	4/4	3/3	5/5
	4.5.9	Ointment may be applied to the wound if indicated.	4.50/4.50	4.5/4.5	4/4	5/5	4.56/4.50	5/4.5	4/4	5/5
	4.5.10	When opening the bag with the large gauze, put on another pair of clean gloves, and you can hold the corner of the gauze and place the center of the gauze over the wound to cover it.	4.25/4.13	4/4	3/3	5/5	4.56/4.31	5/4	4/3	5/5
	4.5.11	Adhere the gauze over the wound with tape. Remove the gloves and drop them into a trashcan. Finally, wash and clean your hands.	3.88/4.00	4/4	3/3	5/5	4.19/4.13	4/4	3/3	5/5
	4.6	If you are allergic to tape, a low-allergy tape or bandage can be used to manage the wound.	4.19/4.25	4/4	2/3	5/5	4.19/4.19	4/4	2/3	5/5
	4.7	Observe the wound carefully; tell your doctor or other professionals and visit the clinic as soon as possible if								
	4.7.1	Redness is noted over or around the wound.	4.50/4.63	5/5	3/4	5/5	4.56/4.69	5/5	3/4	5/5
	4.7.2	The yellowish or green discharge has a bad odor or more discharge is noted from the wound.	4.44/4.94	5/5*	3/4	5/5	4.63/4.94	5/5	3/4	5/5
	4.7.3	Bleeding is noted again or cannot bestopped even with ten minutes of direct pressure.	4.63/5.00	5/5*	3/5	5/5	5.00/5.00	5/5	5/5	5/5
	4.7.4	Swelling or pain is noted around the wound.	4.19/4.50	4/4.5*	3/4	5/5	4.69/4.69	5/5	3/4	5/5
	4.7.5	The skin edge of the wound breaks over 0.5 cm.	4.31/4.31	4/4	3/3	5/5	4.56/4.50	5/4.5	4/4	5/5
	4.7.6	The skin edge of the wound remains wet.	4.13/4.44	4/4.5*	3/3	5/5	4.50/4.44	5/4.5	3/3	5/5
	4.7.7	Your body temperature is elevated over 38.5°C.	4.56/4.88	5/5*	4/4	5/5	4.75/4.81	5/5	4/4	5/5
	4.7.8	You have any questions concerning the condition of the wound or its care.	4.19/4.63	4.5/5	2/4	5/5	4.50/4.50	5/4.5	2/4	5/5

*p < 0.05

### The pilot study

During the study period, 30 eligible trauma patients presenting to the ED were approached and completed the questionnaire. The baseline characteristics of the participants who completed the questionnaire are provided in Table 4.

**Table 4:** Baseline characteristics of the participants in the pilot study

Characteristic	No.	%
Age		
< 20	6	20.0
20–29	9	30.0
30–39	7	23.3
40–49	2	6.7
50–59	3	10.0
> 60	3	10.0
Gender		
Female	16	53.3
Male	14	46.7
Education		
≤ High school	14	46.7
College	16	53.3

The distributions of the correct responses on each question on the knowledge measure before and after watching the educational video are provided in Table 5. The top 13 questions were chosen according to their ranking by the experts, and one question was replaced because it was correctly answered by more than 85% of the subjects when the knowledge measure was piloted on 10 subjects. Two questions were further eliminated because the differences in the correct response rates before and after watching the educational video were not statistically significant in the pilot test. The final knowledge measure comprised 10 questions that were weighted equally and scored.

**Table 5:** Distribution of correct responses to each question

Question	Before Correction rate (%)	After Correction rate (%)	*p*-value
1. The purpose of the debridement surgery is to (1) relieve pain, (2) examine the infective pathogen, (3) remove dead tissue and foreign bodies from the wound, or (4) all of the above.	36.7	60.0	0.016
2. Which of the following is a risk for surgical debridement? (1) The vessels, tendons, or nerves might be injured, (2) bacteria from the skin might affect the deep tissue and cause infection, or (3) both of the above	56.7	80.0	0.016
3. Which of the following might increase the risks of the procedure and postoperative complications? (1) Using painkillers, (2) using steroids, or (3) using antibiotics.	43.3	80.0	0.007
4. Which of the following conditions might increase the risks of the procedure and postoperative complications? (1) Imbibing alcohol, (2) smoking, (3) drinking coffee, or (4) chewing betel nuts.	10.0	43.3	0.002
5. The appearance of the wound should be observed postoperatively. Which of the following is normal? (1) Redness over or around the wound. (2) The yellowish or green discharge has a bad odor or more discharge is noted from the wound. (3) The body temperature is 37°C. (4) The skin at the edge of the wound remains wet.	26.7	53.3	0.039
6. When after injury should ice packing over the wound be started? (1) 1~3 days, (2) 3~6 days, or (3) longer than 6 days.	66.7	100.0	0.002
7. How long should the ice packing be performed each time? (1) 1~5 min, (2) 10~15 min, or (3) 30~60 min.	46.7	83.3	0.007
8. Which of the following is not the purpose of ice packing? (1) To stop the bleeding, (2) increase circulation, or (3) alleviate pain.	73.3	93.3	0.031
9. When after injury should hot packing be applied? (1) 1st day, (2) 2nd day, or (3) 3rd day or later.	70.0	93.3	0.039
10. If the gauze adheres to the wound, what can you do when changing the dressing? (1) Remove it directly, (2) use hydrogen dioxide to rinse the gauze, or (3) use normal saline to rinse the gauze.	76.7	96.7	0.031

The results and distribution of the knowledge scores before and after watching the educational video are presented in Table 6. A significantly higher mean knowledge score was observed after the participants watched the video than that before they watched the video education. The average knowledge score before watching the video was 55.33, and that after watching the video was 78.33.

**Table 6:** Participant knowledge scores in the pilot study (*n* = 30)

Outcome	Before		After		*P*-value
	Mean	Standard Deviation	Mean	Standard Deviation	
Knowledge score	55.33	18.33	78.33	11.17	0.00

The distribution results of the satisfaction ratings are presented in Table 7. A relatively high percentage of patients expressed satisfaction with the video-based informed consent process. A relatively high percentage of patients indicated that they comprehended the information provided by the video regarding the surgery and that the video helped them make a decision.

**Table 7:** Distribution of satisfaction ratings of the educational video

Outcome	Before No (%)	After No (%)	*P*-value
I can comprehend the information that the healthcare providers provided regarding the surgery			0.00
Strongly agree	7 (23.3)	17 (56.7)	
Agree	19 (63.3)	11 (36.7)	
Fair	3 (10.0)	2 (6.7)	
Disagree	1 (3.3)	0 (0.0)	
Strongly disagree	0 (0.0)	0 (0.0)	
The information that the healthcare providers provided can help me make a decision regarding the surgery			0.00
Strongly agree	9 (30.0)	17 (56.7)	
Agree	19 (63.3)	13 (43.3)	
Fair	2 (6.7)	0 (0.0)	
Disagree	0 (0.0)	0 (0.0)	
Strongly disagree	0 (0.0)	0 (0.0)	
I am satisfied with the informed consent process for the surgery			0.01
Strongly agree	7 (23.3)	18 (60.0)	
Agree	21 (70.0)	12 (40.0)	
Fair	2 (6.7)	0 (0.0)	
Disagree	0 (0.0)	0 (0.0)	
Strongly disagree	0 (0.0)	0 (0.0)	

## Discussion

We successfully developed an educational video to improve trauma patients’ comprehension and satisfaction with the informed consent process in the ED. The video also provided information regarding informed consent for the surgery, and the pilot study revealed that the video showed promising results in providing better information delivery and improved the satisfaction of the trauma patients. To our knowledge, this is the first report using a Delphi technique to collect experts’ opinions and reach consensus on the content of informed consent education and develop an educational video for the informed consent process. This is also the first study to develop such a video for informed consent in trauma patients.

Furthermore, evaluating the patients’ understanding is a very important operational measurement of the success of the informed consent process. The knowledge test developed by the panel of experts in our study had a high face validity and included information that the authors believe the patients need to know before consent should be signed for surgery. The knowledge measure and satisfaction tools were scientifically developed and piloted, and their success was validated.

Including patients in the decision-making process and providing information regarding their concerns is important for achieving informed consent. Kusec et al. noted that patient involvement is essential in the development of informed consent information and in establishing methods for developing educational materials to improve patients’ understanding of procedures [25]. In our study, several patients were included in our panel of experts to provide valuable viewpoints.

Controversy remains regarding how much information should be provided to patients during the informed consent process [26–28]. Although the law mandates that healthcare providers disclose information concerning the procedures, risks, benefits, and alternatives to the patients, the extent to which this information should be disclosed remains a challenging issue. Reasonable personal and professional standards provide healthcare providers with reference guides to deliberate and deliver adequate information to patients; [1, 27–31] however, progress in trauma treatment is proceeding rapidly, [32, 33] and in our opinion, the topic of whether professional standards should appropriately guide healthcare providers is settled.

An international consensus is lacking regarding the development of an adequate informed consent form and the information that should be included in such informed consent documents. In many hospitals, there are written informed consent forms with detailed explanations of the procedures, risks, and alternatives; however, it should not be presumed that each patient understood all information concerning their case. We inspected many informed consent documents and found variation in the content. Some documents were very long, and some were short. The main categories (procedures, benefits, risks/complications, and alternatives) were included, but the content within the categories varied. In particular, the risks were described differently. Some informed consent documents were quite detailed and listed all possible risks and complications, even including complications that are very rare and unlikely. Some informed consent documents described the risks and complications in general terms without an explanation of the degree of risk. Therefore, a universal consensus and a standardized format for informed consent documents for trauma patients may be needed, and further studies in this area are warranted.

Written consent could be considered protection for clinicians and hospitals from litigation rather than as benefitting patients [26, 30]. However, this is not consistent with the core values and principles of informed consent and could likely be harmful to the patient–physician relationship. Physicians and institutions should develop strategies to improve the informed consent process according to the best interests of the patients. In our study, our proposed methodology could be applied to the development of informed consent content for a specific surgery. The development of the content of informed consent should be based on a scientific method that integrates the opinions of various stakeholders with respect to the procedures of individual hospitals or even different cultures in different countries. Institutions can develop informed consent content in reference to their own policies under the principles of ethics and legal regulations. Nations can also develop unique informed consent content adapted to their different cultures.

The Delphi technique secures a “group” consensus using a structured process in which many rounds of interviews are conducted via questionnaires [34–37]. Choosing suitable experts for participation in the study is important for the Delphi technique to succeed. If the chosen experts adequately represent areas that are relevant to the study of interest, content validity may be ensured [38]. In our study, we invited several experts from different fields with a variety of expertise, including trauma surgeons, nurses, informed consent experts, a lawyer, and patients who had previously received debridement surgery, to participate in this Delphi round. In our opinion, the validity of the content was ensured.

The Delphi technique has several advantages. One advantage is that each expert’s opinion is considered equally [35, 36]. Experts may compare their own opinions with those of others’ and reassess topics to shape their values and opinions, which could be revised accordingly. In our study, the experts had different ratings for some items in the second round; however, consensus was reached after comparing their own opinions with those of others in the third round. In our opinion, the Delphi technique is a useful tool for building a consensus regarding the content of informed consent and further helps in developing an educational tool.

Giving written materials to patients before they undergo procedures might increase their knowledge, and the written materials could be useful tools for the patients [14, 39]. However, written material usually requires a patient’s active collaboration and compliance, and transfer of knowledge concerning procedures and the risks to the patient is often limited. Significant percentages of patients do not read consent forms before signing [8, 40]. Using video-assisted methods to educate patients has been shown to result in greater patient satisfaction and improved patient understanding of the procedures and risks [14, 39, 41–48]. Although most studies have focused on elective procedures or surgeries, the problems with patient understanding and information retention can be expected to be greater in emergency settings; therefore, institutions are recommended to develop effective educational tools to foster the informed consent process. In our study, the patients had significantly higher knowledge and satisfaction scores after watching the educational video, and we believe that the developed educational video is an excellent tool for the informed consent process in trauma patients. Institutions and healthcare providers should provide standardized and structured information on informed consent to patients to promote their understanding and satisfaction.

In our study, the participants reported that they could better comprehend the information provided by the video and had significantly higher knowledge and satisfaction scores after watching the educational video. We believe that the developed educational video is an excellent tool for the informed consent process. However, the participants watched the video after they were provided with the standard information orally in our study. The testing of the patients’ knowledge after watching the educational video was performed in participants who were provided this information orally; therefore, the patients’ increased understanding may be due to the combination of both oral and visual presentations of the information. However, the repetition of the information may have also enhanced the patients’ understanding and knowledge. Future studies are needed to evaluate the effectiveness of the educational video as a stand-alone tool or as an adjunct to the traditional informed consent process.

Recent technological advances in portable and tablet computer technologies have provided good opportunities for improving patient education regarding surgery [5]. Because portable computers currently have larger screen displays, larger memory storage, and significantly better image resolution, we can more easily deliver educational information and videos with good quality presentation. Consequently, we believe that the use of innovative portable computer technology may improve preoperative education in trauma patients requiring emergency surgery.

Informed consent is more than a process or a document [26, 29, 49, 50]. According to Manson and O’Neil, “informed consent is sought and obtained by distinctive sorts of communicative transactions” [51]. Although we conveyed the information and attempted to make the consent disclosure more complete, the process of informed consent should not be entirely replaced by technological tools. Informed consent is a vital process for communicating with patients and families and building trust. Healthcare providers may invite patients and their families to share each other’s values, beliefs, and opinions in making the best medical decision to maximize the benefit to the patient.

Our study has several limitations. First, although the experts in this study represented a variety of specialties, it is possible that their opinions might not have reflected the whole picture. Although patients were included in our expert panel, there were only a few, and all patients had a college-level education. Future studies are needed to include more patients with more diverse educational, age, and cultural backgrounds in the expert panel. By including more patients in the sample, it might be possible to obtain more precise information regarding the information sought by patients rather than rely on experts’ opinions of what patients should be told. Moreover, the best expert panel composition remains to be determined. Future studies should explore procedures for determining the ideal expert panel composition. Different expert panel compositions, such as those consisting mainly of patients, healthy people or people in relevant risk groups, should be explored to determine whether a different consensus is reached based on the composition of the panel in future studies. Further studies might also consider including more experts with a broader spectrum of specialties to provide more thorough opinions. Second, the severity of injury in trauma patients varies and might have an influence on their consent process and perception of satisfaction. Future studies are needed to explore these associations. Third, the questions used in the knowledge measure instrument were selected by the experts in the Delphi panel. Thus, the choice of questions for the multiple-choice test should also be considered from the patients’ perspectives because the aim is to test the understanding of information that the patients consider to be important. Future studies are needed to confirm our results. Fourth, experts were invited, and the participants were enrolled using a convenience approach. The pilot study was not a randomized controlled study design, and there might be many confounding variables limiting our inferences. Moreover, the conventional informed consent at our hospital has not been standardized to serve as a stable baseline for comparisons of the variations caused by the additional knowledge gained from the video. Further randomized controlled studies are needed to confirm the effectiveness of the educational video and to compare the use of the video with the routine informed consent discussion with trauma patients in the ED. Finally, the pilot study was conducted at only one institution, and the results might not be generalizable to other institutions.

## Conclusions

We proposed a methodology that can be applied in developing content for informed consent for a specific surgery. The modified Delphi technique is a good method for collecting experts’ opinions and reaching consensus on the content for informed consent and educational materials. The educational video contains sufficient information developed by a scientific method that integrated the opinions of different stakeholders, particularly patients. A knowledge measure instrument to evaluate the understanding of informed consent in trauma patients was developed and validated. The pilot test revealed that the educational video improved the trauma patients’ knowledge and satisfaction in the ED. Thus, the educational video is a useful tool for improving the knowledge and satisfaction of trauma patients in the ED. Institutions should give top priority to patient-centered health care and the development of a structured informed consent process to improve the quality of care.

## Abbreviations

ED: Emergency department
